# Social Media in Pregnancy Care: Exploring Adoption Factors and Digital Healthcare Information Utilization among Expectant Mothers in Ghana

**DOI:** 10.4314/ejhs.v34i1.6

**Published:** 2024-01

**Authors:** Philomina Pomaah Ofori

**Affiliations:** 1 Department of Mobile and Pervasive Computing, Ghana Communication Technology University

**Keywords:** Emotions, Health information, Pregnant women, Social media

## Abstract

**Background:**

The Antenatal Care (ANC) Center is a conventional facility that caters for the prenatal healthcare needs of expectant mothers and ensures proper management by healthcare professionals; however, expectant mothers seek healthcare support from other sources. This study aimed to examine the utilization of social media for healthcare information among expectant mothers in the capital city of Ghana and explore the factors that influence its adoption.

**Method:**

This study employed a non-experimental survey design. The study used a questionnaire to gather data from expectant mothers. Using 580 valid responses, SmartPLS structural equation modeling (SEM) was used to analyze the study model.

**Results:**

The study findings demonstrated the significant influence of performance expectancy of social media (PESM) and facilitating conditions of social media (FCSM) on social media healthcare information usage (SMHLU). The results also revealed that emotional support on social media and perceived vulnerability were influential factors that shaped expectant mothers' choices to use social media for healthcare information. However, the study showed that perceived severity and the relative advantage of social media had no significant effects on SMHIU. Interestingly, FCSM was found to be significantly associated with PESM, emphasizing that social media support enhances performance expectancy.

**Conclusion:**

This study showed that information is important to expectant mothers, which compels them to seek digital healthcare. With these findings, healthcare providers can incorporate digital health services into their ANC service to support women during pregnancy.

## Introduction

Pregnancy is a significant and crucial time in a woman's life, one that is of the utmost importance and profound significance (1). Due to this, pregnant women are motivated to actively pursue knowledge to educate themselves (2). Promoting the dissemination of wellness information to expectant women is critical for ensuring optimal maternal and fetal health outcomes during pregnancy and childbirth (3). It has been established that (4) there is a need for pregnant women to access comprehensive knowledge and information pertaining to pregnancy, delivery, and childcare, given their unique physical and mental states and attendant responsibilities. Prenatal education programs play a pivotal role in providing expectant mothers with the opportunity to acquire indispensable knowledge and competencies across a diverse range of domains pertaining to the health and well-being of both the mother and the unborn child (5). Obstetric care provides pregnant women with the avenue to gain knowledge about healthy behaviors, ultimately lowering pregnancy-related risks (6). Given the importance of accessing information during pregnancy, expectant mothers have increasingly turned to social media platforms as a means of obtaining relevant knowledge and insights (7,8). The internet has introduced a fresh path for engagement via social networking platforms (9), which bring up digital media that comes with a delightful array of dimensions and opportunities for users to explore and enjoy (10). The inherent characteristics of these digital platforms foster user-generated content, online sharing, and interaction within a multitude of communities (11).

The proliferation of digital technologies has underscored the significance of individuals' digital skills, which have become crucial for participating in the digital age (12). The utilization of social media platforms has emerged as a prevalent mode of communication among expectant mothers in search of healthcare-related information and support. A study (13) ascertained that a significant number of expectant mothers adopt health prenatal applications and social media platforms along with their prenatal care regimen, which has become a customary aspect of the overall pregnancy experience. As stated (14), expectant mothers have gained novel insights regarding their physical state and parenthood as a result of their engagement with social media. A similar study (15) supported the idea that pregnant women utilize digital networking sites not only for healthcare information but also for emotional support. A report (16) posits that expectant mothers utilize digital networks to obtain information on various healthcare matters, such as personal and infant care, infection prevention and control, vaccinations, and other related topics. Moreso, a recent study (17) shows that expectant mothers' trust and satisfaction with digital healthcare information affect their decision to embrace social media health information.

The authors (18) conducted a study in Sweden to explore the use of digital platforms as a source of information during pregnancy. In Iran (19), a study was conducted to assess the usage of the internet among pregnant women. In a study (7), the authors examined pregnancy-related advice shared on social media. Moreover, in India (20), researchers investigated the practice of disseminating pregnancy-related information on social media. Although some studies have been conducted in other jurisdictions, the generalization of the findings from these studies cannot be assumed to be applicable in Sub-Saharan Africa. The present study aims to investigate the determinants that motivate pregnant women in Ghana to embrace social media for their healthcare needs.

**Hypothesis (H)**: Patients have been utilizing social networking sites for health purposes since the early 2000s (21), and these platforms continue to be promising avenues for improving health, especially among historically underserved communities (22). Research (23) shows that social media has emerged as a possible platform for sharing health information. The perceived relative advantage of using digital platforms among patients is a crucial factor that plays a significant role in determining the level of adoption and utilization of such platforms, and it is important to further explore the specific benefits that patients perceive as most valuable in order to enhance their engagement and satisfaction with these platforms (24). In the context of expectant mothers' healthcare information seeking, the “relative advantage of social media” refers to the perceived degree to which the use of digital platforms provides more timely and advantageous benefits compared to conventional healthcare sources. Consequently, the ensuing hypothesis is posited, i.e., H1: The relative advantage of social media (RASM) has a significant positive effect on social media healthcare information usage.

The acceptance of mobile health services by users is significantly influenced by individuals' expectations of the systems' performance (25). Performance expectations and the intention to act based on the information provided are found to be positively correlated (26). Specifically, the presence of facilitating conditions (FC) has been shown to have a statistically significant effect on individuals' likelihood of adopting health information technology (27). Further research suggests that FC has an immediate and significant influence on future technology adoption (28). However, a study found that FC does not affect older people's interest in using mobile health services (29). From the above discussions, “facilitating conditions of social media” is defined as the degree to which pregnant women perceive that there is technical support for the use of social media platforms. Also, “performance expectancy of social media” refers to the level at which expectant mothers perceive that the design and functionality of a digital platform will empower them to gain healthcare knowledge necessary for effective self-management. Again, the author defined “social media healthcare information usage” as a digital platform that provides healthcare information that is utilized by pregnant women for pregnancy management. With the above review, the ensuing hypotheses are posited:

H2: Performance expectancy of social media (PESM) is positively associated with social media healthcare information usage.

H3: Facilitating conditions of social media (FCSM) is significantly associated with social media healthcare information usage.

H4: FCSM has a significant effect on the performance expectancy of social media.

The provision of emotional support can enhance the recipients' perception of their caregivers through the promotion of positive emotional states among those seeking healthcare (30). Prior research investigating digital health platforms for medical care has revealed that offering patients emotional assistance is the most critical element in facilitating their progress towards improved health outcomes (31). Based on this study, “emotional support on social media” is defined as the level at which pregnant women perceive that the digital platform offers a great opportunity for them to receive care and attention from other members. Consequently, the ensuing hypothesis is posited, i.e., H5: Emotional support on social media (ESPSM) has a significant influence on social media healthcare information usage.

Research findings have indicated that perceived severity and vulnerability have a stronger influence on inducing adaptive behavior compared to the benefits of maintaining the current state (32). This implies that threat appraisal plays a significant role in motivating individuals to take proactive actions. In this study, “perceived vulnerability” is defined as the pregnant woman's evaluation of the possibility that there is a potential risk during pregnancy. “Perceived severity” is also defined as the level of intensity of the pregnancy risk on the life of pregnant women. Consequently, the following ensuing hypotheses are posited:

H6: Perceived vulnerability (PV) is positively associated with social media healthcare information usage.

H7: Perceived severity (PS) has a direct positive influence on social media healthcare information usage.

## Material and Methods

**Research design**: To collect the data for this study, a survey instrument employing a convenience sampling technique was utilized. The study commenced in May 2022 and concluded in August 2022. The research instrument used was a questionnaire. This hospital-based research was carried out within the Greater Accra Region of Ghana, with a specific focus on a carefully selected group of hospitals. The establishments included in the study were the Police Hospital, Trust Mother and Child, St. John's Hospital and Fertility Centre, Pentecost Hospital Madina, and Lekma Hospital. The inclusion of these hospitals in the study was based on the expectation that they would attract a significant number of pregnant women seeking adequate antenatal care (ANC) services.

**Study respondents and sampling**: The researcher opted for a convenience sampling approach in this study due to the expectation that a readily available pool of pregnant women would be accessible at the selected hospitals for participation in the survey. The respondents involved in this research comprised pregnant women aged 18 years or older, possessing the ability to read and write. Respondents were informed about the study, and all who were willing to participate were given an information sheet to read. After they were convinced to be part of the study, a consent sheet was given to each participant to sign. After signing the consent form, participants were given the questionnaire to fill out. All the respondents in this study were registered and received ANC at one of the hospitals that were used for the study. The questionnaire was prepared in an anonymous manner, so no personal details were taken.

**Study instruments and measures**: The model for the study consists of seven variables, namely, PESM, FCSM, RASM, ESPSM, PS, PV, and SMHIU, which had 25 items. The study utilized a 5-point Likert scale ranging from one, which indicated strongly disagree, to five, which indicated strongly agree, to assess the respondents' responses regarding the constructs examined. The collected data were analyzed using SmartPLS version 4. The measurement items of PESM, FCSM, and SMHIU were adapted from (33) and relative advantage from (34). Also, the emotional support measurement items were from (35). Meanwhile, perceived vulnerability (PV) and perceived severity (PV) items were adapted from (36).

**Data analysis**: In testing each construct for its effect, structural equation modeling (SEM) was employed in this study. A study shows that (37), SEM can be used to measure how variables predict the effects of other variables. Since SEM has been proven to be effective in validating constructs, it is normally used to test hypotheses. A total of 580 valid responses were used to assess the SMHIU model, which exceeded the recommendation (38). SmartPLS software was used to explore the causal relationship between constructs in the SMHIU model by assessing the measurement and structural models.

**Ethical considerations**: The Ghana Health Service Ethics Review Committee (GHS-ERC) (GHSERC:001/01/22) approved the study, and in addition to ethical approval from the GHS-ERC, all hospitals agreed for their facilities to be used for the study. Again, all respondents gave their consent to be part of the study.

## Results

**Demographic results**: The socio-demographic results show that 107 of the expectant mothers were 18-25 years of age, while 260 were 26-30 years of age. Quite a number of them, 207, were between the ages of 31-40, with only 6 being 41 years of age or older. See Table 1 for the rest of the demographic findings of this study.

**Table 1:** Socio-demographic results (n=580)

Variables and Category	Frequency	%
Age Group		
18-25	107	18
26-30	260	45
31-40	207	36
41 +	6	1
Educational Level		
High School	246	42.4
Diploma	119	30.5
Degree	177	20.5
Postgraduate	19	3.3
Other	19	3.3
Pregnancy Stage		
1st Trimester	151	26
2nd Trimester	224	39
3rd Trimester	205	35
No of Pregnancy		
First Pregnancy	256	44.1
Second Pregnancy	231	39.8
Third Pregnancy	37	6.4
Fourth Pregnancy +	56	9.7

**Measurement model results**: The measurement was based on all the variables. The outer loadings of the items were in the range of 0.632 to 0.915. Again, average variance extracted (AVE) values ranged from 0.518 to 0.673, Cronbach's alpha values ranged from 0.760 to 0894, and composite reliability ranged from 0.767 to 0900. Discriminate validity was assessed using the Heterotrait-Monotrait ratio (HTMT), and the values were from 0.243 to 0.748 (39). Multicollinearity was assessed to eliminate the potential confounding effects of collinear independent variables. The detection of a variance inflation factor (VIF) value exceeding 3.3 indicates that there is multicollinearity (40).

**Figure 1:**
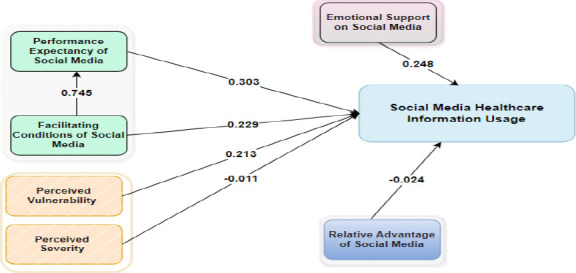
Results of measurement model

In this study, the values obtained for the VIF were in the range of 1.000 to 2.801, indicating that common method bias was not a prominent concern within the scope of this study. Figure 1 shows the path results of the constructs. The standardized root mean square residual (SRMR) was used to check the model fit, and the value was 0.043. The value obtained for SRMR was within the recommended threshold (41,42).

**The structural model findings**: The structural model was assessed using structural equation modeling (SEM). The analysis of the study revealed that PESM (β =0.303; t = 2.835; p < .005), FCSM (β = 0.229; t = 1.987; p <.047), ESPSM (β =0.248; t = 4.066; p <.000), and PV (β = 0.213; t = 3.614; p <.000) had a significant effect on social media healthcare information usage (R^2^ = 0.559). Furthermore, PS (β = -0.011; t = 0.196; p <.845) and RASM (β = -0.024; t = 0.410; p <.682) had an insignificant effect on social media healthcare information usage (R^2^ = 0.559). Moreover, the facilitating conditions of social media (β = 0.745; t = 14.732; p <.000) had a significant effect on PESM (R^2^ = 0.556). The Q^2^ value for PESM was 0.349, and that for SMHIU was 0.391.

## Discussion

This study investigated the factors that influence expectant mothers to use digital information during pregnancy. The findings in this study showed that pregnant women from the ages of 26 to 30 demonstrated a high interest in using digital healthcare information. Interestingly, 42.4% of the respondents had a high school education, which underscores their desire to learn about their condition. Moreover, 44.1% of them were expecting their first child, which is a period of uncertainty, and as a result, it sparks interest to know more about their baby. Out of the number, 39% were in their 2^nd^ trimester, which is a moment to understand and know how to manage oneself.

The results of the investigation into the correlation between the relative advantage of social media (RASM) and SMHIU indicate that the relative advantage of social media has no noteworthy impact on SMHIU, despite initial expectations. The results of the study indicate that the relative advantage of using social media does not increase pregnant women's likelihood of using digital platforms. The findings suggest that when pregnant women require important information at a point when their clinical professionals are not available, they are less likely to resort to social media platforms. The results of this study diverge from related research on the correlation between relative advantage and digital adoption (43), which was conducted in China to assess healthcare facility employees' intent to adopt mobile health services. The findings of the study conducted in China posit that relative advantage has a significant impact on digital health adoption. The study done in China corroborated another study conducted in Saudi Arabia on electronic healthcare.

Moreover, the study revealed a substantial and favorable impact of the performance expectancy of social media (PESM) on the adoption of social media healthcare information usage (SMHIU). This discovery demonstrates how the functionality of digital platforms enhances the inclination of pregnant women to utilize virtual platforms to obtain crucial healthcare information. The current finding confirms healthcare-related studies conducted in Ghana, Malasia, and India (44–46).

The innovative aspect of investigating the impact of facilitating conditions on social media performance expectancy was found to be significant. The results suggest that the joint effect of facilitating conditions and performance expectancy enhances the relationship between the two variables. The outcomes of this study are at odds with the earlier work (47), but in accordance with another research study (48).

The impact of facilitating conditions of social media (FCSM) on social media healthcare information usage (SMHIU) was investigated. The findings revealed a positive association between FCSM and SMHIU. The observed significant relationship between the facilitating conditions of social media and SMHIU may be attributed to the fact that expectant mothers possessed basic knowledge of how to handle technical issues, which enhanced their usage of digital platforms. The current findings are in line with previous research that was conducted in Malaysia (49).

The study measured pregnant women's adoption of social media for healthcare information by utilizing the emotional support on social media (ESPSM) construct. The outcome shows that ESPSM had a significant and positive impact on social media healthcare information usage (SMHIU), which revealed the vital role of psychological support during pregnancy.

The study highlights the vital role of emotional support in expectant mothers' well-being, and given that social media offers an avenue to receive such support, pregnant women are more likely to utilize these platforms to meet their needs. The results of the present study are consistent with healthcare-related research conducted in Ghana (50).

The results indicate that perceived vulnerability has a significant impact on social media healthcare information usage, thus confirming Hypothesis 6. This outcome suggests that expectant mothers are more likely to use social networking platforms for healthcare information when they perceive themselves to be vulnerable to a particular threat. This conclusion aligns with a comparable study conducted in China on health information (36). However, it contradicts another study done in Malaysia (51).

In contrast to prior research, the current study found no significant correlation between perceived severity (PS) and the adoption of social media healthcare information (SMHIU) by pregnant women. This result was unexpected, given that perceived vulnerability was found to have a significant influence on SMHIU. The present study supports the findings of similar studies (52). The findings of the current study are an indication that not all pregnant women rely on social media for healthcare information in the face of serious challenges during pregnancy.

Although this study has made valuable contributions to the field of social media healthcare information, it is important to acknowledge its limitations. The study sample was limited to Ghana, which restricts its generalizability to other countries. As a result, future studies should incorporate other countries to provide a broader understanding of the topic.

In conclusion, the present study utilized the SMHIU model to examine the healthcare information usage of expectant mothers and employed SmartPLS to identify and validate the factors affecting their adoption of digital health information. The results indicate that PESM, FCSM, ESPSM, and PV are significant predictors of social media healthcare information adoption. However, RASM and PS showed no effect on SMHIU. Although this effect was minor, it advances the study in another sphere for future research. Meanwhile, the study highlights the importance of digital platform performance, system support, and emotional support in motivating expectant mothers to use digital media for health information and the role of anticipated threats during pregnancy in driving their engagement with digital health.

These findings offer valuable insights for all healthcare professionals who use social media to connect with expectant mothers. This will enable them to develop effective strategies to encourage the use of healthcare information from reputable sources.

## Figures and Tables

**Table 2:** Reliability and validity results

Constructs	Number of items	Average variance extracted (AVE)	Cronbach's alpha (a)	Composite Reliability (CR)
Emotional support on social media	3	0.650	0.844	0.859
Facilitating conditions of social media	3	0.518	0.760	0.767
Performance expectancy of social media	4	0.547	0.828	0.831
Perceived severity	5	0.620	0.894	0.900
Perceived vulnerability	3	0.560	0.786	0.810
Relative advantage of social media	3	0.643	0.841	0.847
Social Media Healthcare Information Usage	4	0.673	0.892	0.892

**Table 3:** Results of the hypothesis test

Hs	Hypothesis	Path relations	T-statis	p-values	Remarks
H1	RASM -> SMHIU	-0.024	0.410	0.682	Rejected
H2	PESM -> SMHIU	0.303	2.835	0.005	Accepted
H3	FCSM -> SMHIU	0.229	1.987	0.047	Accepted
H4	FCSM -> PESM	0.745	14.732	0.000	Accepted
H5	ESPSM -> SMHIU	0.248	4.066	0.000	Accepted
H6	PV -> SMHIU	0.213	3.614	0.000	Accepted
H7	PS -> SMHIU	-0.011	0.196	0.845	Rejected

*Abbreviations: RASM = relative advantage of social media, PESM = performance expectancy of social media, FCSM = facilitating conditions of social media, ESPSM = emotional support on social media PV = perceived vulnerability, PS = perceived severity, SMHIU = social media healthcare information usage, Hs = Hypothesis*
